# Cytokines (IL1β, IL6, TNFα) and serum cortisol levels may not constitute reliable biomarkers to identify individuals with post-acute sequelae of COVID-19

**DOI:** 10.1177/17562864241229567

**Published:** 2024-02-12

**Authors:** Michael Fleischer, Fabian Szepanowski, Anne K Mausberg, Livia Asan, Ellen Uslar, Denise Zwanziger, Lothar Volbracht, Mark Stettner, Christoph Kleinschnitz

**Affiliations:** Department of Neurology and Center for Translational and Behavioral Neurosciences, University Medicine Essen, University Duisburg-Essen, Essen, Germany; Department of Neurology and Center for Translational and Behavioral Neurosciences, University Medicine Essen, University Duisburg-Essen, Essen, Germany; Department of Neurology and Center for Translational and Behavioral Neurosciences, University Medicine Essen, University Duisburg-Essen, Essen, Germany; Department of Neurology and Center for Translational and Behavioral Neurosciences, University Medicine Essen, University Duisburg-Essen, Essen, Germany; Department of Neurology and Center for Translational and Behavioral Neurosciences, University Medicine Essen, University Duisburg-Essen, Essen, Germany; Department of Endocrinology, Diabetes and Metabolism, Clinical Chemistry – Division of Laboratory Research, University Medicine Essen, University Duisburg-Essen, Essen, Germany; Central Laboratory, University Medicine Essen, University Duisburg-Essen, Essen, Germany; Department of Neurology and Center for Translational and Behavioral Neurosciences, University Medicine Essen, University Duisburg-Essen, Essen, Germany; Department of Neurology and Center for Translational and Behavioral Neurosciences, University Medicine Essen, Hufelandstr. 55, Essen 45147, Germany

**Keywords:** biomarker, immuno-profile, post-acute sequela of COVID-19, SARS-CoV-2

## Abstract

**Background:**

Post-acute sequelae of COVID-19 (PASC) comprise a broad spectrum of symptoms such as fatigue, general weakness, compromised attention and sleep or anxiety disorders. PASC represents a medical and socio-economic challenge.

**Objectives:**

Our study evaluated cytokines (IL-1β, IL-6 and TNFα) and cortisol levels in a cohort of typical patients with PASC, suffering concentration problems, fatigue and difficulties finding words.

**Design:**

This was a prospective cohort study. Four groups were analysed and compared: those who had never contracted SARS-CoV-2 (*n* = 13), infected but had no PASC (*n* = 34), infected with former PASC that resolved (*n* = 40) and patients with ongoing PASC after infection (*n* = 91).

**Methods:**

Cytokine and cortisol serum levels were determined in patients’ blood samples.

**Results:**

Cytokine levels of IL-1β, IL-6, TNFα and cortisol levels did not differ between groups analysed.

**Conclusion:**

This may indicate a non-organic/psychosomatic genesis of PASC; further studies are needed to elucidate the underlying causes of PACS, and non-organic causes should not be overlooked.

## Plain language summary

Without clear biological markers for people who will continue to present with post-acute sequelae of COVID-19 (PASC) should we now focus on psychological factors?

Many people across the globe are still suffering from post-acute sequelae of COVID-19 (PASC), commonly called post-COVID. Typical symptoms of PASC include severe tiredness (fatigue), concentration deficits (brain fog) or difficulty finding words. We need a better understanding of how these symptoms arise to find ways to help patients. Our team of researchers set out to explore this. We posed the question: could measurements of immune system activity provide an identifier for people who are susceptible to post-COVID?

The participants in our study were divided into four groups: 1. A group of 13 people who had never contracted SARS-CoV-2. 2. A group of 34 people who had been infected with SARS-CoV-2 but had no PASC. 3. A group of 40 people who had been infected with SARS-CoV-2 and had already suffered from PASC that had now resolved. 4. A group of 91 people who were no longer sick with COVID-19 but were still suffering from PASC.

Serum samples from all participants were taken to measure cytokine and cortisol levels. People with PASC could not be identified by testing their blood samples for cytokines (IL-1β, IL-6, TNFα) or cortisol. No difference between the four groups was found on any marker. Measuring these cytokines or cortisol is, therefore, unlikely to be useful in predicting which patients will suffer from PASC. Continuation of symptoms long after COVID-19 has passed is distressing for many people worldwide. Psychological factors may play a role and need to be studied further in order to help this patient population.

## Introduction

Post-acute sequelae of COVID-19 (PASC) – for example, fatigue, palpitations, attention, sleep and anxiety disorders^1–3^ – are thought to affect up to 10% of hospitalized patients, albeit recent studies pointing towards significantly lower incidences, in particular since the emergence of the omicron virus variant.^4^ Despite the acceptance that PASC has precipitated significant medical and socio-economic problems, the underlying causes of PASC are yet unclear. Multifactorial origins for these symptoms are being explored, and psychosomatic factors, viral persistence, autoimmunity and a persistent inflammatory response have all been suggested as potential mechanisms.^5^ Understanding PASC pathophysiology, and the identification of biomarkers could be clinically valuable – particularly in predicting the risk of progressing to PASC.^6^

A broad variety of potential aetiologies and biomarkers have already been proposed in PASC.^7,8^ For example, it has been suggested that an increase in α2-antiplasmin may lead to microclots and impaired fibrinolysis in individuals with PASC.^9^ Moreover, increased platelet activation and vascular endothelial dysfunction may be involved in the condition.^5^ Finally, the persistence of SARS-CoV-2 could induce microbiota dysbiosis, autoimmunity and immune priming.^5^ Hence, the spectrum of suggested mechanisms is broad,^10^ as well as suggested surrogate markers of PASC, such as antibodies.^11^ Interesting biomarkers that offer a convenient measurement technique and are potentially compatible with suggested PASC pathophysiology have recently been described, namely the cytokine panel IL-1β (Interleukin-1β), IL-6 (Interleukin-6) and TNFα (tumour necrosis factor alpha),^12,13^ decreased cortisol levels and immune profiling.^14,15^ If the pathophysiology of PASC is based on an excessive immune reaction or aberrant hormone release, then determining these parameters could provide a viable biomarker.

As we previously have not yet been able to find any laboratory abnormalities, obvious immunological changes, increased inflammation or clinical cues of a cortisol deficiency in our PASC patients,^16^ we have strived to reproduce cytokine signatures and altered cortisol levels in a cohort of PASC patients.

## Methods

### Study design and cohort

This study was conducted following the ethical principles of the Declaration of Helsinki. Informed written consent was obtained from all participants.

In total, *n* = 178 participants were analysed in this study, 130 participants fulfilled the WHO Delphi consensus criteria for PASC^17^ and were included. A previous positive PCR (polymerase chain reaction) test had confirmed SARS-CoV-2 infection in all participants. All individuals were recruited from the post-COVID-19 outpatient centre at the Department of Neurology, University Medicine Essen, Germany, between January 2021 and March 2023. In addition, *n* = 13 participants without previous SARS-CoV-2 infection were recruited. To ensure that the group of patients who had never contracted SARS-CoV-2 had no previous exposure to the SARS-CoV2-free status of the control group, antibody testing was performed in five cases, while we relied on assessing past medical history in eight cases.

Participants were stratified into four groups: those who had never contracted SARS-CoV-2 (*n* = 13); those who had been infected with SARS-CoV-2 but did not experience PASC (*n* = 34); those who had been infected with SARS-CoV-2 and experienced PASC that resolved over time (*n* = 40) and those with ongoing PASC post-COVID-19 (*n* = 91).

### Cortisol levels and cytokine concentration in serum

All serum samples were collected between 8.00 and 11.00 a.m. between January 2021 and March 2023. Serum cortisol levels were determined with the Siemens Atellica^®^ IM Analyzer (Siemens Healthineers, Erlangen, Germany). The Atellica^®^ IM Cortisol assay is a competitive chemiluminescence immunoassay with a detection limit of 13.80–2069.25 nmol/l, an intra-assay variation of 7.7% and an inter-assay variation of 2.7%. The instrument controls were performed according to the product inserts (manufacturer’s quality control). Serum cortisol is accredited according to DIN EN ISO 15189:2014. LEGENDplex Human B Cell Panel (13-plex, BioLegend) was used to determine serum IL-1β, IL-6 and TNFα cytokine levels.

### Statistics

Differences for multiple groups were analysed using non-parametric Kruskal-Wallis-ANOVA with Dunnett’s multiple comparison tests after testing parametric distribution with the Shapiro-Wilk test. Pearson’s correlation was used to analyse cytokine and cortisol levels. All statistical analyses were done by SPSS (IBM Corp. Released 2020, IBM SPSS Statistics for macOS, Version 27.0; IBM Corp., Armonk, NY, USA). Graphs were drawn using GraphPad Prism (version 9.5.1 for macOS; GraphPad Software, San Diego, CA, USA). The level of significance was determined by *p* < 0.05.

## Results

### Demographics

Demographics between the four groups (those who had never contracted SARS-CoV-2, those who had been infected with SARS-CoV-2 but did not experience PASC, those who experienced PASC that resolved and those with ongoing PASC) were comparable, although patients in the group ‘no prior COVID-19’ were significantly younger, compared to the average age (*p* = 0.02).

Patients with preceding COVID-19, in most cases, experienced a mild to moderate course of infection (mild: 54.8%, moderate: 43.4%, severe: 1.8%) according to the severity scale implemented by Buonsenso *et al*.^17^ Of those participants who reported PASC (*n* = 131), the mean severity was 3.4 ± 2.5, according to the severity score established by Bahmer *et al*.^18^ in the COVIDOM study. PASC patients presented with a median duration of the symptoms 7 ± 7.4 months after the acute infection (Table 1). The most common symptoms reported were deficits in concentration (67.8%), fatigue (39.2%) and difficulties finding words (14.7%). There was no difference in comorbidities across the groups (data not shown). One patient was infected with SARS-CoV-2 despite preceding vaccination.

**Table 1.** Demographic characteristics of studied chohorts and disease-related parameters for SARS-CoV2 infection.

Parameters	Total	No prior COVID-19	Never PASC	Resolved PASC	Ongoing PASC
Number	178	13	34	40	91
Age					
Mean ± SD (years)	44.5 ± 13.9	33.4 ± 11.8	38.7 ± 16.3	46.6 ± 12.9	47.3 ± 12.6
Sex					
Female (%)	66	63	65	61	66
Severity of COVID-19					
Mild (%)	54.8		73.5	42.5	51.2
Medium (%)	43.4		26.5	55.0	46.6
Severe (%)	1.8			2.5	2.2
Hospitalization for acute infection (days)	3 (1.6%)	n.a.	0 (0%)	2 (5.0%)	1 (1.0%)
Time infection to study inclusion (months)	7 ± 7.4	n.a.	n.a.	5.5 ± 4.9	7.0 ± 8.1
Severity of PASC (severity score, Bahmer *et al*.)	3.4 ± 2.5	n.a.	n.a.	2.2 ± 1.2	3.8 ± 2.6

PASC, post-acute sequelae of COVID-19.

### Serum cytokines

**Figure 1.**
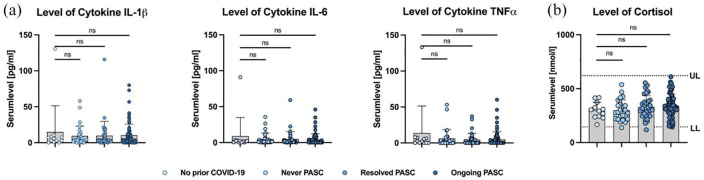
(a) Serum levels of IL-1β, IL-6 and TNFα (mean ± SD). (b) Serum levels of cortisol (mean ± SD) with upper limit (UL, 620 nmol/l) and lower limit (LL, 145 nmol/l) of normal. ns, not significant.

No differences between the stratified groups could be found for the cytokine levels of IL-1β, IL-6 and TNFα (Table 2; Figure 1). The three cytokines positively correlate within individuals. The strongest correlation was detected between IL-6 and TNFα (*r* = 0.89), followed by IL-1β/IL-6 (*r* = 0.64) and IL-1β/TNFα (*r* = 0.45, data not shown).

**Table 2.** Levels of cytokines IL-1β, IL-6 and TNFα and levels of cortisol levels according to COVID-19/PASC status.

Parameters	Total	No prior COVID-19	Never PASC	Resolved PASC	Ongoing PASC
Number	165	12	29	39	85
Cytokine (pg/ml)					
IL-1β (mean ± SD)	10.4 ± 18.4	14.9 ± 36.5	9.6 ± 13.6	9.7 ± 19.8	10.3 ± 15.5
IL-6 (mean ± SD)	4.7 ± 10.8	8.9 ± 25.9	4.8 ± 8.1	4.7 ± 10.4	4.1 ± 8.0
TNFα (mean ± SD)	5.6 ± 14.4	13.7 ± 37.7	6.0 ± 12.7	4.5 ± 8.7	4.8 ± 10.4
Number	142	12	24	34	72
Cortisol (mmol/l)					
Mean ± SD	339.7 ± 141.3	298.4 ± 76.1	298.7 ± 97.7	331.8 ± 105.2	340.5 ± 112.6

PASC, post-acute sequelae of COVID-19.

### Serum cortisol

No difference in serum cortisol levels between the groups was detected and serum cortisol levels were within the normal range in all groups (Table 2; Figure 1). No correlation was identified between individual participants’ serum cortisol levels and cytokine concentration (data not shown).

## Discussion

In this study, cytokine levels of IL-1β, IL-6 and TNFα or cortisol levels did not show suitability as biomarkers to identify or objectify PASC. A prior study by Schulteiß *et al.* showed that after 8 months post-acute infection, patients with ongoing PASC show cytokine dysregulation. In particular, the triad of IL-1β, IL-6 and TNFα was identified to correlate with the presence of symptoms.^13^ Another study found reduced cortisol levels associated with PASC *versus* non-PASC.^14^ Surprisingly, however, cytokine and cortisol levels did not differ between the groups in our study. This is despite similar study populations concerning the distribution of demographic characteristics, the compatible spectrum of PASC and the use of the same methods and testing kits. However, the relatively small sample size and the unequal sample distribution of the four analysed groups must be regarded as limitations of our study.

Several reasons could account for the discrepancy in findings; for example, concomitant diseases in the participants could partially explain formerly reported higher cytokine levels. Bronchial asthma is associated with heightened cytokine levels^19^; analogously, these patients had higher cytokine levels in our study. Therefore, these conditions must be considered when interpreting cytokine levels in general. However, in the study by Schultheiß, a detailed characterization of the participant’s comorbidities is not provided.^13^ Cortisol levels had no diagnostic value in identifying PASC and showed a broad inter-patient variability. Different pretest conditions might affect the variability of cortisol levels, such as the time of the blood draw and concomitant diseases.

Previous studies have suggested various biomarkers in PASC,^8,15,20^ but small sample sizes and lack of cohort stratification may limit some of these studies. Therefore, caution is advised concerning drawing a broad conclusion from studies with moderate sample sizes, unadjusted risk factors or unmeasured characteristics.^21^

In summary, the above-mentioned cytokines and cortisol are not appropriate biomarkers. The results of this study are consistent with our previous findings and those of others who did not find any laboratory changes and have suggested a non-organic/psychosomatic genesis of PASC.^16,21,22^ Further studies are necessary to elucidate the pathophysiology of PASC^10^ but non-organic causes should not be overlooked.

## Declarations

**Ethics approval and consent to participate:** The study was approved by the local ethics commitee of the University Duisburg-Essen, Germany. Reference numbers 20-9284-BO, 20-9307-BO.

**Consent for publication:** Not applicable.

**Availability of data and materials:** The data that support the findings of this study are available from the corresponding author, CK, upon reasonable request.
